# Herpes Simplex Encephalitis: Detection, Management, and Outcomes

**DOI:** 10.7759/cureus.31962

**Published:** 2022-11-28

**Authors:** Melanie N Rayan, Raghav Bassi, Maher Khazem, David A Pozo, Wael Abduljaber, David B Burtis

**Affiliations:** 1 Internal Medicine, University of Central Florida College of Medicine, Graduate Medical Education/North Florida Regional Medical Center, Gainesville, USA; 2 Radiology, University of Florida Shands, Gainesville, USA; 3 Internal Medicine, University of Central Florida College of Medicine, Graduate Medical Education/HCA Florida North Florida Hospital, Gainesville, USA; 4 Neurology, HCA Florida North Florida Hospital, Gainesville, USA

**Keywords:** kluver-bucy syndrome, auto immune encephalitis, temporal lobe encephalitis, herpes simplex virus infection, temporal lobe seizure, herpes virus encephalitis, herpes encephalitis

## Abstract

The pathophysiology of herpes simplex encephalitis (HSE) is incompletely understood and proposed to be secondary to the retrograde transport of the herpes simplex virus type 1 (HSV-1) via the trigeminal and/or olfactory nerves to the central nervous system (CNS). In this case report, we present a 68-year-old female who presents to our emergency department after a fall. Upon initial admission, her neurological examination was benign, and a computer tomography (CT) scan of her brain showed a subdural hematoma for which she was treated conservatively. Day 4 of her hospitalization marked a rapid decline in her course of illness, beginning with confusion and hallucinations, progressing to subclinical seizures, and culminating in irreversible brain damage and palliative extubation on day 16 of hospitalization. This case report discusses our insight into the challenges of early diagnosis and treatment of herpes encephalitis and their impact on improving patient outcomes.

## Introduction

Herpes simplex virus type 1 (HSV-1) encephalitis is a severe complication of HSV-1 infection, accounting for approximately 10-20% of fatal encephalitis cases annually in the United States [1]. Globally, it is estimated that two-thirds of adults under the age of 50 are infected with HSV-1 [1]. Of those infected, herpes encephalitis is estimated to affect 2-4 individuals per 1,000,000 per year worldwide [1]. Due to the broad differential diagnosis, including encephalitis from other viral etiologies or post-infectious conditions, brain tumors, and paraneoplastic and autoimmune encephalitis, among others, the diagnosis can be quite challenging, particularly in an acute setting. Clinical manifestations of HSV-1 encephalitis include a febrile prodrome, headache, altered mental status, focal neurologic deficits, new-onset seizures, and behavioral changes. It is a necrotizing hemorrhagic inflammatory encephalitis with a propensity for affecting the mesio-temporal lobes. The pathophysiology is incompletely understood; however, proposed mechanisms include retrograde transport of the virus via the trigeminal and/or olfactory nerves to the central nervous system (CNS) [2]. Early recognition and treatment with acyclovir or a similar agent are pivotal and have been shown to improve patient prognosis [3-5]. Early, aggressive antiviral therapy can prevent mortality and reduce the severity of chronic post-encephalitic cerebral impairments such as Klüver-Bucy syndrome [6]. We present the case of a 68-year-old female who presented after a fall with a subdural hematoma and eventually developed herpes simplex encephalitis (HSE), leading to neurological demise. The case provides insight into the challenges of early diagnosis and treatment of the disease and how it can lead to better patient outcomes.

## Case presentation

A 68-year-old woman with a past medical history of hypertension, hyperlipidemia, and type 2 diabetes mellitus presented to the hospital after experiencing a syncopal episode with loss of consciousness commencing with a fall at home. She regained consciousness en route to the hospital and did not appear confused upon initial presentation. The episode was not preceded by aural symptoms or lightheadedness, and no post-ictal confusion was observed. She denied any prior episodes of syncope or seizures. She endorsed a week's history of flu-like symptoms, including headaches, chills, and diffuse body aches in the week preceding the syncopal episode. She denied neck pain or rigidity, fevers, weakness, or focal neurological deficits. She denied taking any anticoagulation at home. Physical examination was notable for pain on palpation of the posterior aspect of the head and right hip bruising and tenderness, all of which were attributed to the fall.

**Figure 1:**
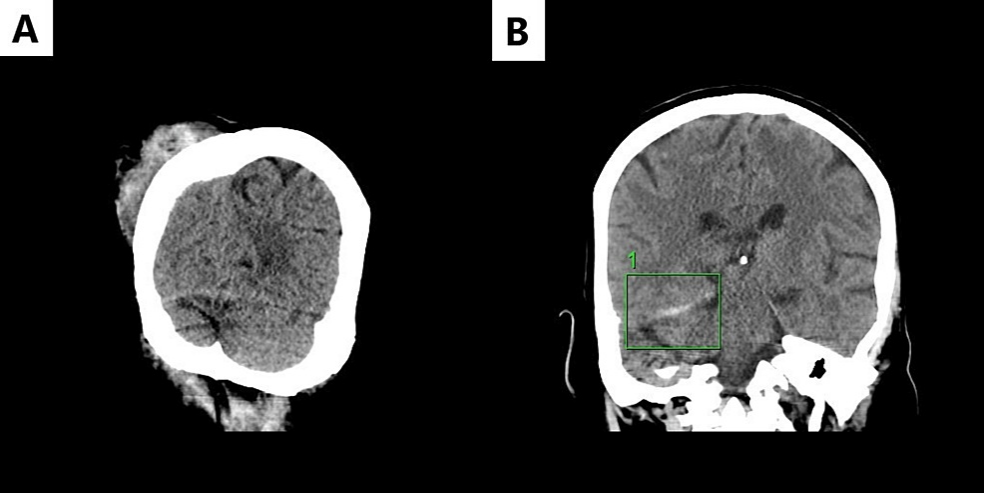
(A) Initial CT after fall showing large posterior paramedian subgaleal hematoma; (B) initial coronal CT showing small 5 mm subdural hematoma along the right tentorium CT: computer tomography

Vitals upon initial presentation included blood pressure (BP) of 194/79 mmHg and tachycardia of 120 beats per minute (bpm). Initial lab work was notable for a white count of 11.2 × 10^9^/L with neutrophilic predominance, sodium of 127 mEq/L, and glucose of 215 mg/dL. The patient was pan-scanned to evaluate for potential injuries secondary to the fall. A computed tomography (CT) of the lumbar and cervical spine was normal; however, a 15-mm right upper lobe nodule was incidentally discovered, for which an outpatient CT imaging of the chest was recommended. CT brain without contrast showed a large right posterior paramedian subgaleal hemorrhage without an underlying fracture, as shown in Figure 1A. A 5 mm-thick subdural hemorrhage was seen along the tentorium without midline shift, as shown in Figure 1B. The patient was treated conservatively, and her systolic blood pressure (SBP) was kept at <140 mmHg. The work-up for syncope returned negative, including a transthoracic echo.

During the initial three days of hospitalization, the patient experienced intermittent low-grade fevers, an elevation in her blood pressure, and tachycardia. She also endorsed a dull headache and paresthesias in her left leg. She was evaluated with a computed tomography angiography (CTA) of the chest, which was negative for a pulmonary embolus. Blood cultures were obtained, and antibiotics were initiated.

Day 4 of hospitalization marked a significant change in her clinical course. Symptoms of confusion and hallucinations along with unstable vital signs prompted evaluation with a repeat CT brain, which showed improvement in the subdural hematoma. Soon after her CT, she became acutely aphasic and non-verbal, and a stroke alert was called. The neurological exam was notable for an awake patient who was unable to follow commands and showed no response to verbal or painful stimuli. Her pupils were equal and reactive. She exhibited a forward gaze and was unable to track visually. A decreased tone in her left upper extremity was noted. Her GCS score was 8. Upon further examination, she developed a left-sided gaze, though she was still able to cross midline along with lip-smacking movements. Due to suspicion of a seizure, she was loaded with 1500 mg of levetiracetam along with 2 mg of lorazepam. The stat electroencephalogram (EEG) revealed intermittent sustained seizure activity noted to be bilateral with left-sided predominance, indicating active subclinical status epilepticus (SCSE). She was started on a propofol and midazolam drip, intubated, and transferred to the intensive care unit (ICU). Long-term EEG monitoring showed approximately two hours of intermittent sustained seizure activity, with complete resolution of SCSE after initiation of the above-mentioned anti-epileptic regimen.

**Figure 2:**
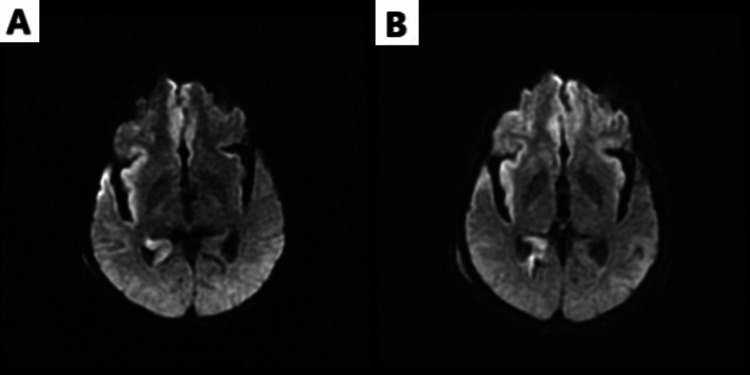
(A) MRI day 5: DWI showing mild diffusion restriction and cortical edema along the mesial frontal mid convexities, right insular cortex, right anterior temporal lobe, and right hippocampus without vasogenic edema or hemorrhage; (B) MRI day 8: DWI showing worsened restricted diffusion with subtle associated meningeal enhancement corresponding to the restricted diffusion most prominently within the right temporal lobe and insular cortex. MRI: magnetic resonance imaging; DWI: diffusion-weighted imaging.

On the morning of day 5, her white count had increased to 21 × 10^9^/L. She was weaned off of the midazolam and propofol overnight, and lacosamide 150 mg twice daily was started. Magnetic resonance imaging (MRI) of the brain without contrast performed on day 5 is shown in Figure 2A. MRA of the head and neck returned unremarkably.

On day 6, a lumbar puncture was performed, which showed colorless cerebrospinal fluid (CSF) with a glucose of 93 mg/dL, a protein of 61 mg/dL, a white count of 66 cells/mm^3^, and a red blood cell count of 61 cells/mm^3^. CSF fluid was sent for analysis, and the patient was started on intravenous (IV) acyclovir at 10 mg/kg every eight hours due to concern for viral encephalitis due to the seizure activity estimated to be in the bifrontal lobes.

Despite being off sedation, the patient remained lethargic for the next two days. Her neurological exam showed involuntary movements in the hand intermittently, raising concern for partial seizures. She was loaded with Fosphenytoin 1000 mg IV, started on a ketamine drip, and doses of lacosamide and levetiracetam increased to 200 mg twice daily and 1500 mg twice daily. Another stat EEG was ordered, which revealed diffuse cerebral dysfunction. Long-term EEG monitoring was initiated, which continued to show burst suppression alternating with focal electrographic seizures intermittently in the left greater than right temporal lobes. Treatment with anti-epileptic agents and acyclovir was continued, and a diffusion-weighted MRI was performed on day 8, which revealed the progression of the disease shown in Figure 2B.

**Figure 3:**
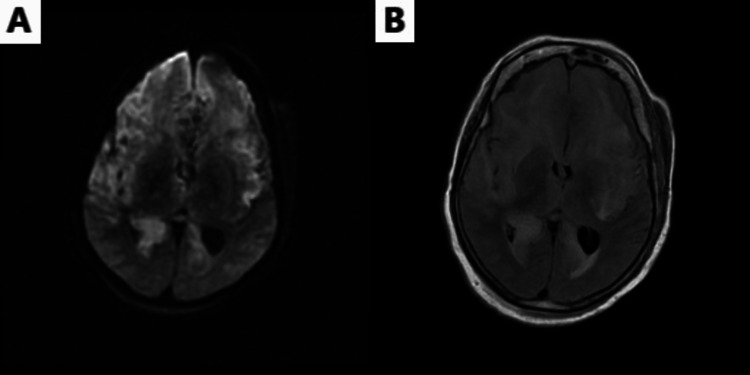
(A) Day 13: diffusion-weighted MRI showing progressive restricted diffusion bilaterally; (B) day 13: MRI T2 imaging showing advanced edematous change involving the bilateral mesial frontal lobes, insular lobes, external capsules, medial temporal lobes, and anterior temporal lobes. MRI: magnetic resonance imaging; T2: transverse relaxation time.

To confirm the suspicion, the HSV polymerase chain reaction (PCR)-1 test of the CSF returned positive on day 11 of hospitalization. Despite having been treated with acyclovir for five days, it appeared that she had no response, as evidenced by her clinical deterioration. Acyclovir was continued, and a repeat CT and MRI of the brain after two days of treatment showed advanced edematous changes and findings suggestive of cytotoxic cerebral edema, shown in Figure 3A-3B. Continuous EEG continued to show significant cortical suppression of activity.

By this time in the patient's course of illness, the prognosis for any meaningful recovery appeared very poor. She was observed for a few days without sedation, with no positive changes in neurological status that may have indicated otherwise. She remained comatose, failed multiple spontaneous breathing trials, and was determined to have irreversible brain damage. On day 16, her family elected to admit her to hospice services for compassionate extubation.

## Discussion

Acute encephalitis is defined by a rapidly progressive inflammation of the brain parenchyma, with symptoms occurring in less than six weeks, and can pose a significant burden on families and patients [4]. The underlying pathophysiology by which HSV-1 affects the CNS in humans is postulated to be due to retrograde transport through the olfactory nerve, which passes directly to the frontal and mesio-temporal lobes, and the trigeminal nerves, which innervate the meninges with subsequent spread to the orbitofrontal and mesio-temporal lobes [2]. Early on in the infectious process, HSV-1 infection triggers activation of toll-like receptors (TLRs), resulting in dimerization of the TLRs, which then initiates a cascade of downstream pro-inflammatory cytokines such as tumor necrosis factor, interferons, and various other interleukins [2]. This inflammatory process activates the innate immune cells and primes the adaptive immune system, resulting in leukocyte recruitment and necrosis and apoptosis of infected cells. After the primary infection, the virus remains in a latent state with the help of HSV-specific CD8+ T cells in the trigeminal ganglia, and deficiencies in this immune response can result in the reactivation of the virus [2]. 

Clinically, the first step in the diagnosis and evaluation of patients begins with a careful medical history and examination which includes: recent illnesses, ill contacts, travel, exposures to animals, vaccination status, chronic diseases/immunocompromised conditions, recent rashes, and hearing loss [7]. Diagnostic criteria for encephalitis include a major criterion that must be seen: altered mental status with a decreased level of consciousness, personality changes for ≥24 hours with no other identifiable etiology, and at least two minor criteria: fever, new-onset seizures, focal neurological symptoms, CSF white blood cell count ≥5 cells/mm^3^, and electroencephalogram changes or neuroimaging changes consistent with encephalitis [3]. When encephalitis is strongly suspected, patients should then undergo CSF analysis through a lumbar puncture if there are no contraindications, and brain imaging through MRI should be obtained urgently [2]. Non-contrast CT scans of the brain are commonly done upon admission but are unlikely to show acute changes in HSE until the fifth day after presentation [8]. MRI imaging, specifically diffusion-weighted imaging (DWI), is the gold standard for identifying lesions caused by HSE [9]. Findings may include asymmetric hyperintense lesions corresponding to areas of edema in the mesiotemporal and orbitofrontal lobes and the insular cortex [9]. It is important to note that PCR analysis for HSV can be falsely negative in the first 72 hours of symptom onset, and patients with a strong clinical suspicion should still be empirically treated with acyclovir with a repeat CSF study in three to seven days [2,3].

Some common distinguishing clinical features that can raise suspicion for HSV encephalitis include olfactory hallucinations and feelings of déjà vu in the early stages due to temporal lobe involvement [7]. HSV encephalitis can also cause limbic encephalitis, resulting in anterograde memory dysfunction, behavioral changes, seizures, headaches, and focal neurological deficits with evidence of temporal lobe involvement in neuroimaging. Other signs of limbic encephalitis include autonomic instability, psychosis, and orofacial dyskinesia, with autoimmune etiologies against the N-methyl-D-aspartate (NMDA) receptor most commonly occurring in patients who have a history of HSV encephalitis, with other infectious etiologies affecting the temporal lobe being ruled out [4]. Antibodies against the NMDA receptor represent a mere 4% of all cases, with behavioral changes being the earliest clinical sign and usually observed in patients with underlying ovarian teratomas [4,7]. Studies have also shown that around 20% of patients with underlying HSV encephalitis can also develop post-infectious autoimmune encephalitis in response to unknown antigens. The underlying pathophysiology is attributed to molecular mimicry, the release of antigenic proteins from neuronal injury that can act as immune targets, and the host's autoimmune response to the HSV infection itself [10]. Another common cause of autoimmune limbic encephalitis is antibodies against the gamma amino butyric acid-B (GABA-B) receptor, with the mainstay of clinical symptoms being a rapid focal neurological decline, acute onset fever, epileptic seizures, orolingual movements, and non-specific respiratory symptoms. It is often seen secondary to paraneoplastic disorders such as small-cell lung cancer, with some cases also being reported in the setting of viral infections [7,11].

As seen in the case presented above, the patient was diagnosed with HSV encephalitis based on serological evidence from PCR tests confirming HSV in the CSF, clinical symptoms consistent with temporal lobe involvement, such as hallucinations, and change in neurological function with radiological evidence of infection in the temporal and frontal lobes. Other differential diagnoses for the patient's encephalitis were also considered, including medication-induced, travel-induced, tick/mosquito exposure, cranial neuropathies, tumors, paraneoplastic encephalitis, and metabolic and progressive multifocal leukoencephalopathy. The patient’s lack of travel history and exposure to ticks/mosquitoes ruled these out as causes of her symptoms. Furthermore, the acute nature of her symptoms ruled out paraneoplastic or tumor-induced encephalitis, which typically has a subacute presentation. She also lacked any symptoms consistent with brainstem involvement, including horizontal conjugate palsy, extraocular movement deficiency, or dysphagia. Imaging was done, and it did not reveal any evidence of progressive multifocal leukoencephalopathy. MRI with and without contrast is the imaging modality of choice, and imaging typically reveals asymmetric hyperintense lesions on T2-weighted images in the mesiotemporal, orbitofrontal lobes, and insular cortex early in the disease process. Temporal lobe involvement is predominantly unilateral; however, recent studies have shown that bilateral temporal lobe involvement is associated with a worse outcome, as seen in the case above.

First-line treatment for patients diagnosed with HSV-1 encephalitis is acyclovir at 10 mg/kg every eight hours for 14 days in immunocompetent patients and 21 days in immunocompromised patients, with the addition of steroids in patients with evidence of cerebral edema or a mass effect [3-5]. Some studies have shown that resistance to acyclovir can develop in patients through a thymidine kinase gene mutation. Although mostly seen in immunocompromised individuals, a few cases have also been observed in immunocompetent patients with successful treatment with foscarnet [5]. Recent guidelines recommend that patients with confirmed HSV-1 encephalitis who do not respond well to antiviral therapy or have recurrent or new neurological symptoms undergo antibody testing for post-infectious autoimmune encephalitis [4,5]. Treatment for post-infectious autoimmune encephalitis includes immunosuppressive therapy with steroids, intravenous immunoglobulins or plasma exchange and evaluation for possible underlying malignancy as treatment of the underlying cancer is the hallmark of therapy in patients [4,12]. A second-line immunosuppression agent such as rituximab or cyclophosphamide can then be added if the patient is still experiencing neurological decline with confirmed evidence of positive antibody testing [4]. Early diagnosis of post-infectious autoimmune encephalitis is crucial because many patients respond well to immunosuppressive therapy and should therefore remain high on the list of differential diagnoses in patients who do not respond well to antiviral therapy, as seen in the case presented above.

## Conclusions

As clinicians, it is essential to carefully assess patients who present with mental status changes (e.g., headaches, focal neurological deficits, new-onset seizures, and altered mental status). HSV encephalitis continues to remain one of the most common etiologies of viral encephalitis in the United States, despite widely available antiviral treatment options. PCR for HSV-1 in the early stages can lead to false negatives, and rapid initiation of acyclovir should be promptly implemented if HSV-1 is suspected in order to prevent permanent neurological damage and even death, as seen in the case presented above.
